# Understanding Protein Functionality and Its Impact on Quality of Plant-Based Meat Analogues

**DOI:** 10.3390/foods12173232

**Published:** 2023-08-28

**Authors:** Jenna Flory, Ruoshi Xiao, Yonghui Li, Hulya Dogan, Martin J. Talavera, Sajid Alavi

**Affiliations:** 1 Department of Grain Science and Industry, Kansas State University, Manhattan, KS 66506, USA; jflory09@ksu.edu (J.F.); rosexiao@ksu.edu (R.X.); yonghui@ksu.edu (Y.L.); dogan@ksu.edu (H.D.); 2 Sensory and Consumer Research Center, Kansas State University, Manhattan, KS 66506, USA; talavera@ksu.edu

**Keywords:** plant proteins, physicochemical properties, animal protein alternatives, structure–function relationships

## Abstract

A greater understanding of protein functionality and its impact on processing and end-product quality is critical for the success of the fast-growing market for plant-based meat products. In this research, simple criteria were developed for categorizing plant proteins derived from soy, yellow pea, and wheat as cold swelling (CS) or heat swelling (HS) through various raw-material tests, including the water absorption index (WAI), least gelation concentration (LGC), rapid visco analysis (RVA), and % protein solubility. These proteins were blended together in different cold-swelling: heat-swelling ratios (0:100 to 90:10 or 0–90% CS) and extruded to obtain texturized vegetable proteins (TVPs). In general, the WAI (2.51–5.61 g/g) and protein solubility (20–46%) showed an increasing trend, while the LGC decreased from 17–18% to 14–15% with an increase in the % CS in raw protein blends. Blends with high CS (60–90%) showed a clear RVA cold viscosity peak, while low-CS (0–40%) blends exhibited minimal swelling. The extrusion-specific mechanical energy for low-CS blends (average 930 kJ/kg) and high-CS blends (average 949 kJ/kg) was similar, even though both were processed with similar in-barrel moisture, but the former had substantially lower protein content (69.7 versus 76.6%). Extrusion led to the aggregation of proteins in all treatments, as seen from the SDS-PAGE and SEC-HPLC analyses, but the protein solubility decreased the most for the high-CS (60–90%) blends as compared to the low-CS (0–40%) blends. This indicated a higher degree of crosslinking due to extrusion for high CS, which, in turn, resulted in a lower extruded TVP bulk density and higher water-holding capacity (average 187 g/L and 4.2 g/g, respectively) as compared to the low-CS treatments (average 226 g/L and 2.9 g/g, respectively). These trends matched with the densely layered microstructure of TVP with low CS and an increase in pores and a spongier structure for high CS, as observed using optical microscopy. The microstructure, bulk density, and WHC observations corresponded well with texture-profile-analysis (TPA) hardness of TVP patties, which decreased from 6949 to 3649 g with an increase in CS from 0 to 90%. The consumer test overall-liking scores (9-point hedonic scale) for TVP patties were significantly lower (3.8–5.1) as compared to beef hamburgers (7.6) (*p* < 0.05). The data indicated that an improvement in both the texture and flavor of the former might result in a better sensory profile and greater acceptance.

## 1. Introduction

Plant-based meat analogues and other alternative protein products have become increasingly popular in recent years as the number of “flexitarian” consumers increases [1]. These consumers are demanding a product that is equally or more nutritious, affordable, environmentally friendly, and tastier than their animal-derived counterparts. With these goals, challenges arise, such as the ability to accurately mimic the texture and other important sensory aspects of a meat product. This research aimed to investigate the impact of protein functionality on texture and to see if functionality information can be used to formulate recipes to target certain applications, such as a plant-based burger or fish filet. Previous research has shown that plants’ protein chemistry and their interactions with water and other physicochemical properties can affect the texture of the final product [1,2,3,4]. Traits and functionalities of plant proteins that are important for the extrusion texturization and quality of plant-based meat analogues include the protein sedimentation coefficient, amino acid composition, least gelation concentration, denaturation temperature, water and oil absorption capacity, viscosity, and flow temperature [2]. Particularly, protein functionality for texturization is highly dependent on protein denaturation and gelling temperatures. The physicochemical properties related to hydration properties of different plant proteins (pea, wheat, and soy) have been analyzed, and their impact on the quality of texturized plant-based meat analog products has been reported [1,3]. Proteins with a high water absorption capacity and cold-swelling properties were found to have greater crosslinking potential and resulted in a porous, less layered internal structure, while proteins with heat-swelling and/or low-cold-swelling characteristics led to a dense, layered extrudate structure. The textural properties of the final product varied depending on protein functionality, emphasizing the importance of understanding and utilizing raw-material properties to achieve desired textural qualities in plant-based meat. The impact of the protein chemistry on the quality characteristics of extruded plant-based meat analogues has also been studied [4]. It was found that the protein composition influenced the structure of meat analogues, although the correlation was not significant. Moderate 11S/7S ratios (1.5:1 to 2.0:1) of soybean proteins led to meat analogues with acceptable nutrition and flavor characteristics, highlighting the importance of selecting soybeans with a consistent 11S/7S ratio for higher-quality end products.

However, protein chemistry and composition are not easy to measure and/or monitor for the purpose of quality control and the design of new products. Moreover, the processing history of plant proteins can further confound their functionality. The plant-based meat industry needs quick tools to fill this gap. Studies have shown evidence that pea protein produces a softer texture than soy and that wheat proteins of gluten can improve texturization [5,6,7]. Previous research by our group has attempted to explain these differences in a systematic manner and showed that cold-swelling proteins have higher crosslinking potential, which leads to an expansion of the texturized vegetable protein (TVP) product, causing a softer and spongier final texture, while heat-swelling proteins lead to a denser and more layered TVP [1,3]. This study focused further on the study of the hydration-related physicochemical properties of various plant proteins derived from soy, wheat, and yellow peas. It was hypothesized that various plant proteins can be categorized as cold swelling or heat swelling and that information can be used to design meat analogues with varying structures, textures, and end-product quality attributes.

Many plant-based meat analogues, such as the ones utilized for this research, are made via low-moisture extrusion processing. The plant protein concentrates or isolates are extruded to form texturized vegetable protein (TVP) that is milled and later combined with other ingredients, such as water, binders, oils, and seasonings, to make a plant-based meat product. In 2019, 48% of plant-based meat products were made using soy protein [1]. Other proteins, such as wheat gluten, pea, and fava protein, are also commonly found in plant-based meat, with the industry beginning to explore a wide variety of protein sources [1,2]. Extrusion utilizes a combination of mechanical and thermal energy to texturize the protein, which means that it realigns the native globular structure of the plant protein into fibrous layers that mimic an animal’s muscle structure. This allows for a more authentic layered structure to be the base of plant-based meat products [8].

Research on improving the texture of plant-based meat analogues through the manipulation of ingredients with different functionalities is a critical step forward in improving the quality and sensory attributes of current plant-based meat. This was one of the goals of this study, and it came with the expectation that this study will pave the way for new, innovative plant-based products to be made targeting previously difficult or unattainable texture goals.

## 2. Materials and Methods

### 2.1. Formulation 

A total of 6 treatments were tested with varying ratios of cold-swelling proteins to heat-swelling proteins (0% to 90% CS or 0:100 to 90:10 CS:HS), as shown in Table 1. Treatments were all soy based (50% or higher), while pea protein and wheat gluten were also used to modulate texture. Proteins characterized as heat swelling included wheat gluten and Arcon-F soy protein concentrate (SPC), and cold swelling included soy protein isolate (SPI), Arcon-S SPC, and pea protein isolate (PPI). Lower-protein or starch-based ingredients, such as soy flour and tapioca starch, respectively, were also used to influence texturization, as they been shown in previous studies to increase layering by interrupting the protein crosslinking that occurs during extrusion processing [9]. The protein content of the treatments were kept between 67 and 78% because a higher protein content can influence the texture of the final TVP by making it tougher and chewier [8]. The formulation details for each treatment can be found in Table 1.

### 2.2. Extrusion Processing 

**Figure 1:**
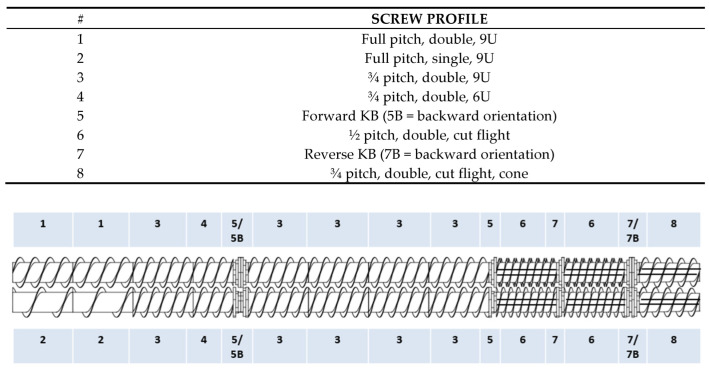
Screw profile for the pilot-scale extrusion trial.

Extrusion processing was performed on a TX-52 pilot-scale twin screw extruder (Wenger Manufacturing, Sabetha, KS, USA) with a barrel diameter of 52 mm and L/D ratio of 19.5. The screw profile used is illustrated in Figure 1. Aggressive screw elements, such as reverse kneading blocks and cut flight elements, were incorporated to increase barrel fill and shear. The extrusion process conditions, including the raw-material feed rate (50 kg/h), barrel temperatures (30, 50, 80, and 100 °C from feed to discharge end), screw speed (300–340 rpm), and in-barrel moisture (42–48% wet basis), were kept constant or adjusted within a narrow range to obtain optimum texturization for each treatment. The exceptions were a lower in-barrel moisture content (38–40% wet basis) for treatments with pea protein isolate and a higher screw speed (449 rpm) for the treatment with the greatest amount of Arcon-F soy protein concentrate. This is discussed further in the Section 3. The target in-barrel moisture was reached by adjusting water injection into the preconditioner and extruder barrel. Steam injection was not used for any of the treatments. A ¼ inch venturi die was used upstream of the final dies to increase shear and mechanical energy and promote texturization. Two outlet dies, each ¼ inch in diameter, were used, and the product was cut after discharge from the extruder, using 3 hard rotating knives. Half of the product was sent directly to a dual-pass dryer (Series 4800, Wenger Manufacturing, Sabetha, KS, USA) after exiting the extruder, while the other half was taken directly off the extruder and milled using an Urschel mill with a screen size of 0.18 inch. Processing information was recorded twice for each treatment at the beginning and end of the collection time and was also collected using a data acquisition system every second.

Specific mechanical energy (SME) was calculated using the following formula:

(1) $$SME\left(\frac{\mathrm{kJ}}{\mathrm{kg}}\right)=\frac{W-W_{0}}{m_{f}}$$

where *W* is the power consumed by extruder (measured using a watt meter), *W_0_* is the power consumed at no load and *m_f_* is dry material feed rate in kg/s.

In-barrel moisture (IBM) content was calculated using the following equation:

(2) $$IBM\left(\%\mathrm{wb}\right)=\frac{\left(m_{f}\times X_{wf}\right)+m_{wp}+m_{we}}{m_{f}+m_{wp}+m_{we}}$$

where *m_f_* is the dry feed rate, *X_wf_* is the moisture content of the dry feed material (expressed as wet basis fraction), *m_wp_* is the water injection rate into the pre-conditioner in kg/h and *m_we_* is the water injection rate into the extruder in kg/h.

### 2.3. Water Absorption Index

The water absorption index (*WAI*) was used to identify cold-swelling proteins by quantifying the amount of water that can be absorbed by a material. The WAI of raw materials was measured for each treatment mix, along with the individual ingredients. The WAI represents cold-swelling abilities because it detects the water absorbed at room temperature, with no thermal energy added. The method used was adapted from a previous study [10]. The test involves mixing 2.5 g of sample with 30 mL of distilled water, using a vortex mixer, for 10 s. The samples were then placed on a shaker table to continue mixing for 30 min. Next, the samples were centrifuged at 3000 g for 15 min, using a Centrifuge 5810 R 15 Amp Version (Eppendorf, Hauppauge, NY, USA). The supernatant was then removed from the sample, as it should separate out from the gel formed at the bottom of the test tube. The gel mass was then recorded. The water absorption index (*WAI*) is calculated by dividing the weight of the total suspension or gel (*W_gel_*) by the dry weight of the precipitated solids (*W_dry solids_*) left after removing the supernatant, as shown in the equation below.

(3) $$WAI\left(g/g\right)=\frac{W_{gel}}{W_{dry\;solids}}$$

### 2.4. Least Gelation Concentration

The least gelation concentration (LGC) was measured for the raw-material blends used in each treatment and the individual ingredients. The LGC is used to identify heat-swelling properties, as it measures the materials’ capacity to form a gel at certain concentrations after undergoing a heat treatment. According to a method adapted from a previously reported study [11], suspensions of raw-material concentrations of 8, 10, 12, 14, 16, 18, and 20% were made using 10 mL distilled water and placed in a series of test tubes. These test tubes were then mixed until the material was completely combined and there were no clumps present. The samples were then placed in a water bath at 90–100 °C for 1 h, followed by a bath in room-temperature water for 10 min. After this was completed, the samples were placed in a refrigerator for another 2 h. After refrigeration was complete, the test tubes were inverted, and observations were then made to conclude if the sample formed a strong cohesive gel at each concentration. The smallest concentration at which the sample did not slip or fall down the sides of the test tube indicated the LGC.

### 2.5. Rapid Visco Analysis

The rapid visco analysis (RVA) was also used to determine the cold- and heat-swelling capabilities and was one of the primary tests used to characterize proteins into either category. A rapid visco analyzer (RVA 4500, Perten Instruments, Waltham, MA, USA) was used to determine the viscosity of the material slurry over time as it was stirred continuously and underwent a heating and cooling cycle. A peak in viscosity at the beginning of the cycle before heating takes place indicates a cold-swelling protein, while a later peak indicates a heat-swelling protein. The samples were prepared by combining the raw materials with distilled water to create a 15% d.b. suspension. The RVA test parameters were set according to the AACC Method 76–21.02 STD1, which involved keeping the sample at 50 °C for 1 min and then heating to 95 °C at 12.2 °C/min, where it was held for 2.5 min. Next the sample was cooled back to 50 °C at 11.8 °C/min and then held for an additional 2 min.

### 2.6. Protein Solubility

The protein solubility was measured to determine how much water the protein fractions of each ingredient and treatment were able to absorb. This can be used to confirm the results found from the RVA, LGC, and WAI to characterize each protein type as cold or heat swelling. To begin, 0.5 g of a sample was dispersed in 10 mL of deionized (DI) water. Once the sample was thoroughly mixed, the original pH was recorded. The mixture was then stirred for 30 min at room temperature to allow for the samples to be fully hydrated. Next, the suspension was centrifuged at 4500 rpm for 30 min. The supernatant was then removed, and the remaining precipitate was frozen and freeze-dried. The precipitate protein content was then tested and used to calculate the protein solubility, using the equation below. The protein content was found using the combustion method (AACC Method 46–30.01) and a LECO analyzer, and a nitrogen-to-protein conversion factor of 6.25 was used. The protein solubility was calculated using the difference between the weights of protein in the original sample and the precipitate, as described in the equation below.

(4) $$Protein\;solubiltiy\;\left(\%\right)=\frac{Protein\;in\;sample\;\left(g\right)-Protein\;in\;precipitate\;(g)\;}{Protein\;in\;sample\;(g)}\times 100$$

### 2.7. SDS-PAGE

Sodium dodecyl sulphate–polyacrylamide gel electrophoresis (SDS-PAGE) was used to determine the molecular weight of proteins present in each ingredient and treatment before and after extrusion. If a decrease in intensity of bands is seen after extrusion, it could indicate polymerization and crosslinking of the proteins [12]. First, 100 mg of each sample was suspended in 10 mL of PBS (pH = 6.8) buffer containing 2% *w*/*v* SDS. The samples were then mixed using a shaker for 1 h (250 rpm) at room temperature, followed by centrifugation at 8000 g for 5 min. The supernatant was then collected and used for the SDS-PAGE analysis, using non-reducing conditions. Non-reducing conditions were chosen for this application to capture the presence of crosslinking because reducing conditions would break down disulfide bonds and split the protein into subunits and not give an accurate depiction of the state of the protein before and after extrusion. To begin the SDS-PAGE analysis, 30 µL of each sample was combined with 10 µL of 4 × Laemmli buffer (Bio-Rad Laboratories, Inc., Hercules, CA, USA). The mixture was then heated in boiling water for 5 min. Following heating, 15 µL of each treatment sample was loaded into wells (10 µL for isolated protein raw materials) of a 4–20% Mini-Protean TGX gel (Bio-Rad Laboratories, Inc., Hercules, CA, USA) and separated at 200 V for around 37 min, all at room temperature. To watch the molecular weight progress, a Precision Plus Protein Dual Color Standards (Bio-Rad Laboratories, Inc., Hercules, CA, USA) was loaded (5 µL) parallelly. A Brilliant Blue R Concentrate (Sigma, St. Louis, MO, USA) was then used to stain the gel, with gentle shaking, for 8 min. DI water was then used to repeatedly de-stain the gel until the background was clear and readable.

### 2.8. SEC-HPLC

SEC-HPLC was also used to detect crosslinking by looking at the presence of certain molecular weights before and after extrusion. Like the preparation for SDS-PAGE, 100 mg of each sample was mixed with 20 mL 2% SDS in PBS (pH = 6.8) and then vortexed for 1 h at room temperature, followed by centrifugation at 8000 g for 5 min. The supernatant was then collected and diluted to 1 mg/mL, using 2% SDS in PBS, and then filtered with a 0.45 µm PVDF membrane filter. Next, the prepared samples were loaded on a Yarra 3 µm SEC-4000 column (300 × 7.8 mm, Phenomoenex, Torrance, CA, USA) and processed using an Agilent HPLC 1100 system. The elution solvents contained water with 0.1% trifluoroacetic acid (A) and acetonitrile (B). The linear gradient was 0 min at 80% A, 20 min at 70% A, 25 min 65% A, and 30 min 80% A. The detection level was set at 214 nm, and the injection volume and flow rate were 20 µL and 0.7 mL/min, respectively. This was conducted at 30 °C.

### 2.9. Water Holding Capacity

The water holding capacity (WHC) was calculated as the amount of water that whole dried extrudate pieces can absorb divided by the initial weight of the sample. First, 15 g of the whole dried extrudate pieces was added to excess water and allowed to soak for 20 min. After the extrudates were fully hydrated, water was allowed to drain, using a strainer, for 5 min, and the WHC was then determined as per a previously reported method [9].

### 2.10. Visual Analysis

A visual analysis was conducted using a Nikon D750 SLR digital camera with a Nikkor 105 mm macro lens to capture several images of the internal structure of hydrated whole extrudate pieces that were cut in two different directions, longitudinal (with the grain) and horizontal (against the grain). Extrudate pieces were hydrated using the same protocol mentioned for WHC. A visual analysis can help to compare and evaluate the changes in texturization and denseness of layering that happens as the material is extruded and crosslinked.

### 2.11. Texture Analysis

A texture analysis was performed using a TA-XT2 Texture Analyzer (Texture Technologies Corp., Scarsdale, NY, USA) according to a previously reported method [9]. A dual compression test was utilized that measures the peak force needed to compress the sample 7 mm twice with a 2-inch-diameter cylindrical probe. This was chosen because it mimics the biting action of the mouth and has been previously used for a variety of meat and meat analogue products [13]. Hydrated pieces were placed one layer deep in a shallow circular dish that was slightly larger in diameter than the probe to contain the sample as it was being compressed. The compression cycles were performed at 1.00 mm/s, and the parameters that were recorded were used to calculate the hardness, chewiness, and springiness [14]. Tests were performed with 10 replicates for each treatment. Plant-based patties (see Table 2 for recipe) were also tested using the texture profile analysis (TPA) protocol adapted from a previously reported study [5]. The patty recipe includes milled TVP, water, oil, seasonings, fava protein concentrate, and methylcellulose as a binder. Plant-based patties were cut into 2″ × 2″ squares. A TPA test was then conducted by compressing the patties twice to 50% of the original height, using the same cylindrical probe, with a speed of 1.00 mm/s. Data from both types of compression testing can be used to calculate the hardness, springiness, and chewiness. Hardness (g) is defined as the peak force during the first compression cycle, and chewiness (g) is the (Area 2nd peak/Area 1st peak) × hardness × springiness, and springiness (mm/mm) is the distance 1st compression/distance 2nd compression [15].

### 2.12. Sensory Analysis 

A focus-group-based study and a consumer study were conducted under Institutional Review Board (IRB) approval #05930 at Kansas State University. All respondents were recruited via email surveys, using Compusense (Compusense Software, Guelph, ON, Canada), from a database of consumers in the Kansas City area maintained by the KSU Sensory and Consumer Research Center, Olathe, KS, USA.

Two 90-min focus-group sessions were conducted to determine consumer perceptions of plant-based meat products and to gain feedback on the texture of experimental plant-based patty products based on low and high CS:HS protein ratios, respectively. The sessions were formed based on whether a low-CS protein product (Group 1) or high-CS protein product (Group 2) was being tested. The two sessions were conducted with a total of 16 participants (8 participants per session, with a total of 11 female and 5 male participants). The participants of the study were aged 18–61 and indicated in a screening survey that they were interested at least somewhat in plant-based protein alternatives. A discussion was conducted following a predetermined discussion guide, focusing first on general perceptions of plant-based meat products and then more specifically on the samples prepared for this study. Each focus-group session tasted a plant-based meat product made from one of the experimental treatments, a commercial plant-based equivalent, and an actual meat product (beef hamburger or fish patty).

A Central Location Test (CLT) or consumer study was also conducted to determine liking differences among treatments and to provide an assessment of how close the test product’s texture was to a real meat product. This was performed based on the assessment of liking and softness/firmness, as described below. Overall-liking, as well as flavor- and texture-liking, ratings were measured using a 9-point hedonic scale, going from “dislike extremely” to “like extremely”. Softness/firmness was measured using a 5-point just-about-right (JAR) scale, going from “much too soft” to “much too firm”. A total of 78 participants aged 18–61 were selected using the requirement criteria that they were at least moderately interested in plant-based protein and were the primary shopper or equally shared grocery shopping for their household. Each person tasted a total of 5 products, which were 4 plant-based treatments and 1 actual beef burger. A completely randomized design was used so that each participant tasted each of the 4 plant-based products in a random order but with the real beef patty always in the last position in order to avoid introducing any bias into the evaluation of the test samples. Participants were asked about overall liking, texture, flavor, aftertaste, and overall perceptions of plant-based meat, using the combination of scales mentioned above and also short-answer questions. For both the focus-group and consumer study, each product was grilled in an electric griddle, using nonstick cooking spray, with an end temperature of 74 °C, and served immediately warm in a 4 oz. plastic cup covered with a lid. The plant-based patty recipes used for both the consumer study and focus groups can be found in Table 2.

### 2.13. Statistical Analysis 

A statistical analysis was completed using SAS software (SAS, Cary, NC, USA) and one-way ANOVA with Tukey’s test to determine the *p*-values and significant differences (*p* < 0.05) and relationships between treatments. For sensory study results, a statistical analysis was conducted on consumer-liking data, using XLSTAT for each attribute, using one-way ANOVA with Fisher’s LSD for pairwise mean separation among treatments.

## 3. Results and Discussion

### 3.1. Extrusion Processing

Through pilot-scale extrusion, native globular plant proteins are hydrated and plasticized in the extruder barrel due to the addition of pressure, shear, and thermal energy. As the material exits the die, the structure of the plasticized material is realigned into a fibrous structure, and disulfide bonds are formed between protein molecules. This is called texturization, and it can cause changes in the conformation and structure of the proteins, along with modifying their functional and physical properties [16]. During processing, one of the most important factors that impacted the quality of the TVP was the die temperature [17]. Different die temperatures were ideal for each treatment (Table 3). Overall, the lower % CS treatments required a higher die temperature, which makes sense because they had a higher ratio of heat-swelling proteins in their formulation that required more thermal energy to form viscosity. The higher % CS treatments had lower die temperatures, and this was most likely due to the high amounts of soy protein isolate (SPI). SPI has previously been reported to produce optimal-texture TVPs at a higher moisture content and lower die temperature (50%, 130 °C) [14]. The water addition in the preconditioner was lowest for treatments containing wheat and highest for treatments containing just soy. This is because soy is more soluble, especially before heat is added, and wheat is difficult to hydrate in the preconditioner because it immediately forms a strong and sticky protein-matrix dough when exposed to water and is best handled in the extruder barrel instead [8]. The extruder screw speed varied for each treatment, with the highest being 30% CS at 449 rpm and the lowest 0% CS at 300 rpm. The 30% CS most likely required such a high screw speed to provide mechanical energy to texturize the product fully because it had the most water added of any treatment, and, therefore, the material had a lower viscosity. The specific mechanical energy (SME) was calculated for each treatment and showed no significant differences between treatments, with the highest being 30% CS at 997.2 kJ/kg and the lowest being 50% CS at 874.8 kJ/kg (Table 3). 

### 3.2. Water Absorption Index

**Figure 2:**
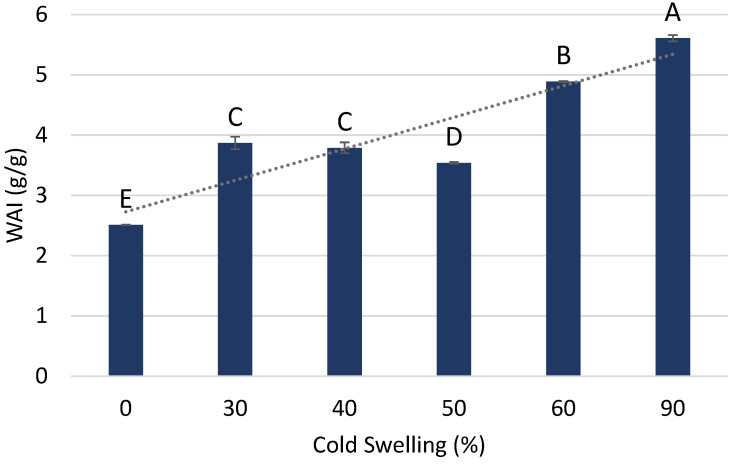
Water absorption index (WAI) for raw-material blends with varying amounts of cold-swelling proteins (0–90% CS). Different letters imply significant differences (*p* < 0.05).

The water absorption index (WAI) was used initially on the raw materials to help characterize the proteins themselves as either cold or heat swelling. A high WAI (>4.0 g/g) indicates cold-swelling abilities, and a low WAI (<4.0 g/g) indicates heat-swelling and/or the absence of cold-swelling characteristics. Proteins that were clearly cold swelling, as indicated by a higher WAI, included soy protein isolate (SPI) and the more functional Arcon-S soy protein concentrate (SPC), at 5.92 g/g and 5.70 g/g, respectively. On the other hand, soy flour and Arcon-F soy protein concentrate had a relatively lower WAI (2.82 and 3.93 g/g, respectively). The WAI was also used to analyze the treatment formulations to further confirm that cold-swelling treatments produced a high WAI and to determine any interactions with heat-swelling components. As the inclusion of cold-swelling proteins in the formulation increased, there was an increasing trend in the WAI as well (Figure 2). The highest WAI was produced by the 90% CS treatment, at 5.61 g/g. On the other end, the lowest WAI was found to be from the 0% CS treatment, at 2.51 g/g. This confirms that the WAI can be used to identify cold-swelling abilities and can help determine the properties of a protein mixture. The WAI of raw materials is dependent on the functionality properties of the proteins which are derived from the subunits and structure that make up each individual protein ingredient. The polarity and availability of different protein residues differ for each source and can impact how the protein interacts with water and its structure. Proteins with lower amounts of polar amino acids may decrease the WAI, and nonpolar may have the opposite effect [16]. For example, the low-cold-swelling characteristics or low WAI of treatments with wheat gluten is at least partly because it comprises prolamins and glutelins that are mostly soluble in alcohols or acids, not in water. The cold-swelling treatments contained soy protein isolate (SPI), which is usually very water soluble, and pea protein isolate (PPI), which is moderately to highly water soluble depending on the ingredient source and isolation method used [1]. Hydrophobicity and, therefore, WAI can also be impacted by different processing methods and steps used during the isolation of the proteins. The commercial production of protein concentrates and isolates often requires the use of physical and chemical methods that modify the functionality of the protein, such as the solubility and folding structure. For example, Arcon F and Arcon S are both soy protein concentrates with similar protein contents (69 and 72%, respectively), but the former is clearly more cold swelling than the latter, as can be seen from the WAI data described above, as well as the RVA and LGC data, which are discussed in the following sections.

### 3.3. Least Gelation Concentration

The least gelation concentration (LGC) was initially utilized to help categorize the raw protein ingredients as either cold or heat swelling. Theoretically, the LGC should be lower for heat-swelling proteins because it takes less material to form a strong gel after heating. Overall, the results of the LGC were not significantly different between treatments when testing both individual protein ingredients and the treatment mixes (Table 4). This is because, although the LGC undergoes a heating cycle to form a gel, some proteins that easily form a gel at room temperature can maintain this state throughout the entire test, until the end, when the results are interpreted. This indicates that cold-swelling proteins that produced a low LGC, such as soy protein isolate, are resistant to shear thinning and can maintain a gel after heating and cooling, which can be a benefit to the final texture of the meat analogue [16]. However, some cold-swelling proteins do not always have good gelling abilities, such as pea protein isolate, and this could be why the treatments were not showing the expected LGC results. Many commercial pea protein isolates are extensively denatured during the isolation process used during production, thus decreasing their gelation abilities and protein–protein interactions [18]. The production of ingredients could potentially be modified to produce a pea protein isolate with better gelation abilities, but it would take more time and less intensive processing.

Overall, it was concluded that the LGC was not the most useful analytical method when determining the swelling properties of plant-proteins; instead, other tests, such as the WAI and RVA, should be relied upon for the characterization of swelling abilities. However, the LGC can still be used to identify differences in protein functionality, and the use of the LGC to identify chemical and functionality differences other than solubility should be investigated further. Gelation may be separate from swelling ability and is another important parameter that needs to be understood further to control the texture and structure of plant-based meat analogues. Gelation is needed to form the final fibrous structure of a meat analogue so that the LGC can indicate the ability to form layered structures in the final product [1].

### 3.4. Rapid Visco Analysis

The rapid visco analysis (RVA) was used to determine the viscosity over a heating cycle with continuous stirring. The RVA was first used to characterize proteins as cold or heat swelling through peaks in viscosity. The peaks in viscosity that occurred before 4 min into the heating cycle were categorized as having cold-swelling properties while peaks later than 4 min were indicative of heat-swelling properties. Multiple peaks may be present, indicating the presence of both the cold- and heat-swelling ingredients. The cold-swelling proteins also had higher peak viscosities, a result which aligns with the WAI data, as the more water that is absorbed, the higher the viscosity that is formed, as was also found in a previous study [16]. This is because cold-swelling proteins such as soy and pea are globular, and this allows them to easily form gels. As they unfold due to shear or heat and are exposed to water, their non-polar and sulfhydryl groups readily form hydrophobic and disulfide bonds that cause the proteins to aggregate and form an increase in viscosity [15].

**Figure 3:**
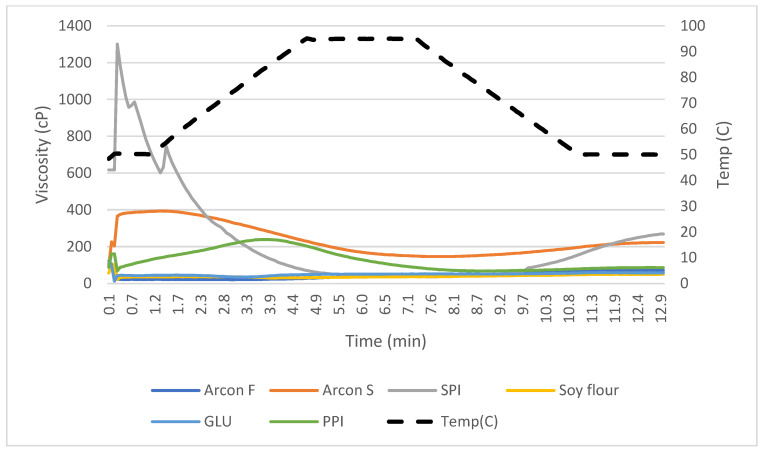
Rapid-visco-analysis (RVA) viscographs for individual ingredients.

**Figure 4:**
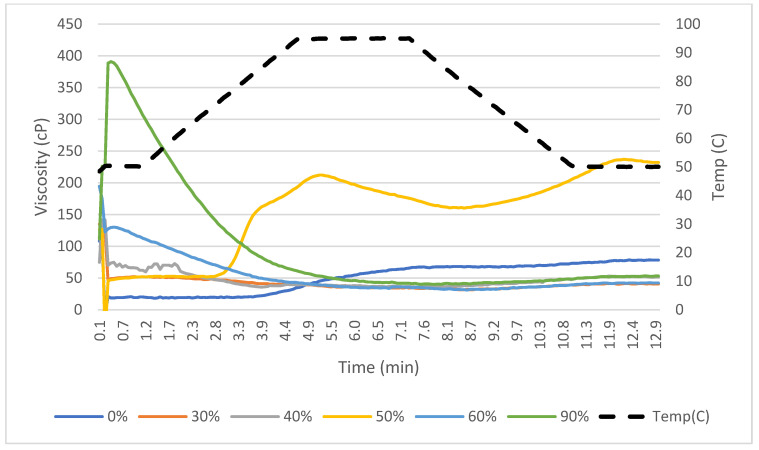
Rapid-visco-analysis (RVA) viscographs for raw-material blends with varying amounts of cold-swelling proteins (0–90% CS).

There were limited statistically significant differences between % CS treatments in peak viscosity. The RVA viscosity curves are shown in more detail for each treatment mix in Figure 3 and Figure 4. The peak time was significantly different for the 90% vs. 0% CS protein mixes, as it was much higher for the heat-swelling treatment because it took more time for the protein to solubilize and increase in viscosity as the heating cycle occurred over time (Figure 4). One factor influencing the lack of significant differences between the peak viscosities of the mixes is that some proteins, such as SPI and Arcon S, are very good at solubilizing and forming a gel, as shown by the LGC data and confirmed by previous studies [15]. This means that even when they were incorporated in smaller amounts, they still overpowered the viscosity peak results of the RVA test. The starch in the formulation also could have had an impact on the viscosity, as starch has a heat-induced peak in viscosity as it is gelatinized [9].

### 3.5. Protein Solubility

The protein solubility for each of the raw-material ingredients helped to characterize and confirm cold-swelling or heat-swelling properties. Overall, a protein solubility (%) of greater than 20% often indicated cold swelling, while less than that indicated heat-swelling abilities. Solubility has previously been shown to be an indicator of protein texturization, with higher solubility leading to more texturization [16]. Soy protein isolate (SPI) had the highest average protein solubility of 58.86%, which was expected and indicates strong cold-swelling properties because the material can solubilize well without heating. Wheat gluten had the lowest protein solubility (14.20%), as was also expected and allowing is to confirm the previous characterization of the protein as heat swelling based on the RVA and WAI data. Wheat gluten also contains the most sulfur-containing amino acids compared to other ingredients, and this could result in greater disulfide bond formation and crosslinking during texturization that would greatly reduce protein solubility [16]. These results align with previously reported solubility data on commercial TVP samples from different protein sources [16]. According to the previous research, soy-based TVP had the highest protein solubility, followed by pea and then wheat [16]. Soy flour had a high protein solubility average of 46.9%, which indicates cold-swelling properties, but it has a much lower protein content, so the effect of starch is more relevant. Starch gelatinizes and increases in viscosity as heating occurs, so the activity of the starch would be considered, such as heat-swelling properties. Previous studies have shown that flour usually increases density and acts more like a heat-swelling protein when used in concentrations less than 20% [9]. The protein solubility decreased overall after extrusion for each treatment (Table 5). This is an indicator of crosslinking because of the increased aggregation of proteins after denaturation and unfolding through the shear and thermal energy of extrusion [16,19].

### 3.6. SDS-PAGE

**Figure 5:**
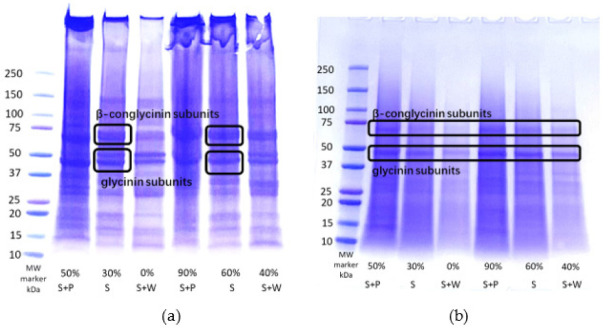
Results from SDS-PAGE analysis, showing bands corresponding to different protein sub-units for treatment with varying amounts of cold-swelling proteins (0–90% CS) both before (**a**) and after (**b**) extrusion.

**Figure 6:**
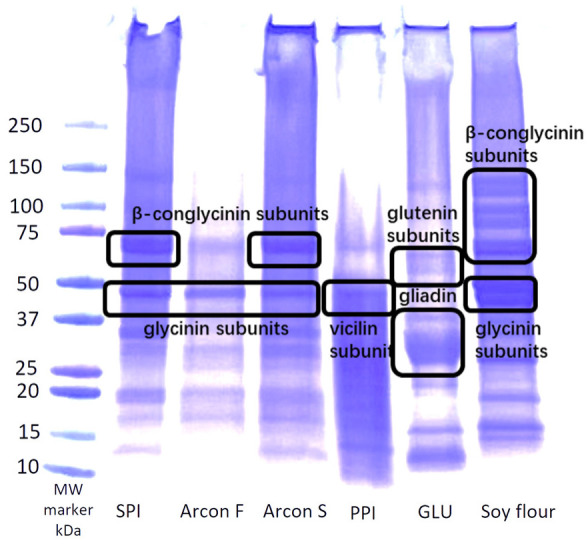
SDS-PAGE results, showing the molecular weights of different protein subunits from individual ingredients.

SDS-PAGE using non-reducing conditions was run on treatment samples before and after extrusion (Figure 5 and Figure 6). Non-reducing conditions were chosen to preserve the bond formation that occurred during extrusion so that the aggregation of protein molecules could be observed. Although these conditions were used, the SDS buffer still breaks apart some of the proteins into subunits. The bands on the gel showed a significant decrease in intensity after extrusion. This is an indicator of polymerization and crosslinking, as shown by a decrease in solubility [12,14,19]. Crosslinking occurs during extrusion, using high heat and shear to denature the proteins and realign them to form new covalent and non-covalent interactions. When the molecules form disulfide bonds and other non-covalent bonds after extrusion, they aggregate to become large enough that they do not pass through the gel and become insoluble, so they are not seen in the same concentrations as before extrusion [20].

Several bands could be seen in the raw-material blends, including those corresponding to β-conglycinin subunits and glycinin subunits (Figure 5a). However, after extrusion, the only prominent bands seen for each treatment were at 75 and 50 kDa, while other bands disappeared or diminished (Figure 5b). This was an indicator of protein aggregation or crosslinking due to extrusion, except in the case of at least part of the soy β-conglycinin subunits and glycinin subunits [21]. This could be because each treatment was soy based, and so there is a higher concentration of soy proteins left even after extrusion. The treatments containing wheat had the most diminished band intensity overall, meaning that they most likely had the most crosslinking and polymerization occur. This is because wheat gluten crosslinks very well due to the higher amounts of sulfur-containing amino acids that are available to form disulfide bonds. Pea protein also has sulfur-containing amino acids in its legumin subunit that are attributed to disulfide bond formation [1]. The Maillard reaction from the heat and shear during the extrusion process can also cause some crosslinking [22]. Heat-swelling treatments required a higher die temperature, which could have increased the Maillard reactions and, therefore, decreased the solubility more than the cold-swelling treatments as well [14]. The treatments containing pea protein would also be affected more significantly by the Maillard reaction because pea protein has more lysine that can bond with glucose during extrusion [14]. The differences in the band intensity between different protein sources can also be explained by a difference in protein solubility that was shown previously. The SDS-PAGE gel showing the individual protein ingredient solubility according to the intensity of the bands is shown in Figure 6. Soy protein often has high solubility, followed by pea and then gluten accordingly. This aligns with the WAI and RVA results that show a decrease in peak viscosity and water absorption as the % CS is decreased. Overall, heat-swelling proteins should show a lighter band intensity, and cold-swelling proteins should have a higher band intensity. This aligns with the results found previously that show a relationship between cold soluble proteins and stronger band intensity [1].

### 3.7. SEC-HPLC

**Figure 7:**
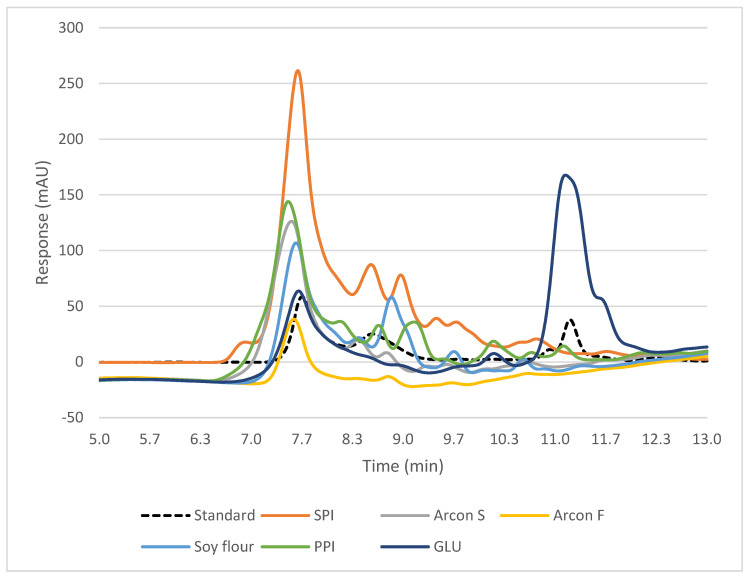
HPLC results for raw ingredients, showing peaks for protein fractions at different molecular weights.

**Figure 8:**
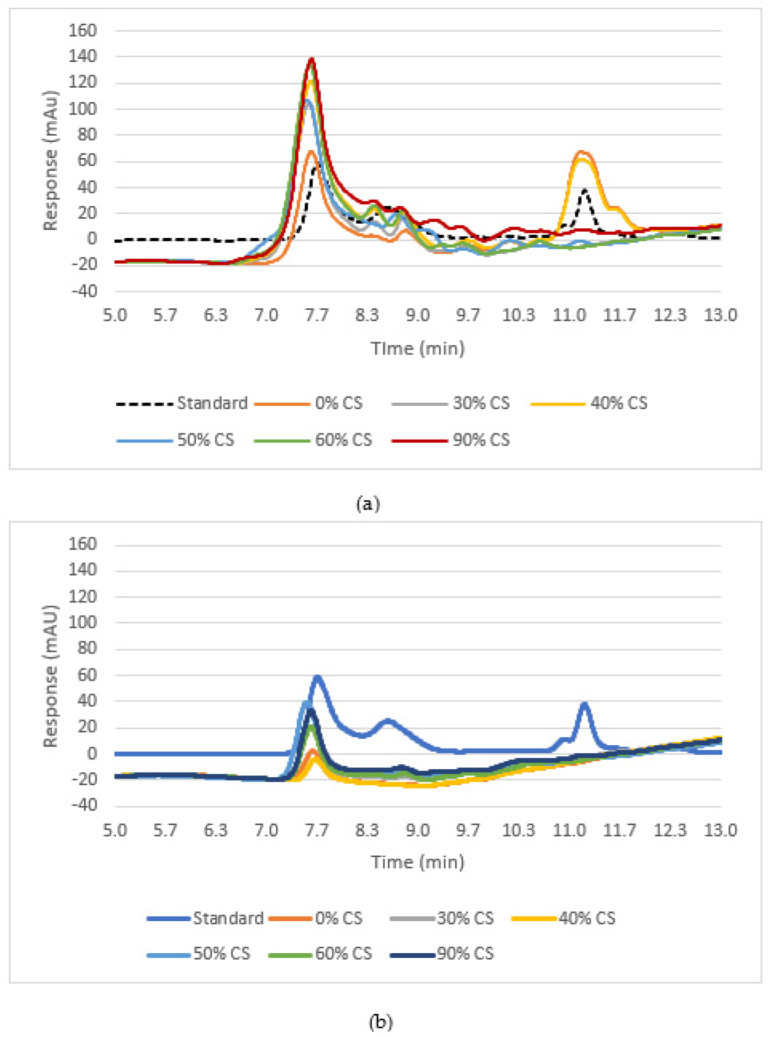
HPLC results for formulations with different cold-swelling protein concentrations (0–90% CS), showing peaks (representing protein fractions) before extrusion (**a**) compared to after extrusion (**b**).

SEC-HPLC was used to confirm the results obtained from the SDS-PAGE, and it also allows for a wider range of molecular weights to be captured and a clearer representation of protein concentration through the peak area rather than band intensity, which is subjective. HPLC also does not use the same buffer as SDS-PAGE, so the proteins are left more in their aggregated native state versus being broken down into their subunits. The peak height represents the concentration of soluble protein present at that molecular weight. A mixture of proteins with known molecular sizes, including thyroglobulin bovine (670 kDa), γ-globulins from bovine blood (150 kDa), bovine serum albumin (60 kDa), and chicken-egg grade VI albumin (44 kDa) (Sigma-Aldrich, St. Louis, MO, USA), was used as the standard and analyzed under the same chromatography conditions to estimate the molecular weight of various proteins’ fractions in individual ingredients and treatment blends, as described previously [23]. The standard peak at 7.52 represents the concentration of molecules at 670 kDa, the peak at 8.77 represents 150 kDa, and the peak at around 11.28 represents 44.3 kDa (Figure 7 and Figure 8). When looking at the individual protein ingredients, we noted that a peak height greater than 0.8 mAU indicated a cold-swelling protein, while a peak less than 0.8 mAU indicated heat-swelling properties (Figure 7). All the peaks for each treatment were either reduced or completely disappeared after extrusion, meaning that all the proteins were successfully texturized and that the proteins were able to aggregate and form disulfide bonds, so they were no longer small enough to be detected (Figure 8). The extrusion process caused the proteins to be less soluble because of the crosslinking that occurred, and, therefore, the peaks were found to be smaller [19]. The peaks that did remain after extrusion for each treatment were at the same molecular size of 670 kDa. This is bigger than most protein fractions, and the large molecular size is most likely from multiple protein molecules interacting and being aggregated together through protein bonds. As mentioned before and as confirmed by the SDS-PAGE results, heat-swelling proteins, such as wheat gluten, readily form disulfide bonds and are more inclined to crosslink, followed by pea and then soy [1,14]. This inclination for crosslinking and disulfide-bond formation comes from the presence of more sulfur-containing amino acids and reactive amino acids such as cysteine, lysine, and glutamic acid [14]. Since soy and pea are slightly less crosslinked and had such a high solubility to start out with, it would make sense that they would still have some solubility remaining after extrusion and be present in small peaks for each treatment, as all contained some soy.

### 3.8. Water Holding Capacity and Bulk Density

**Figure 9:**
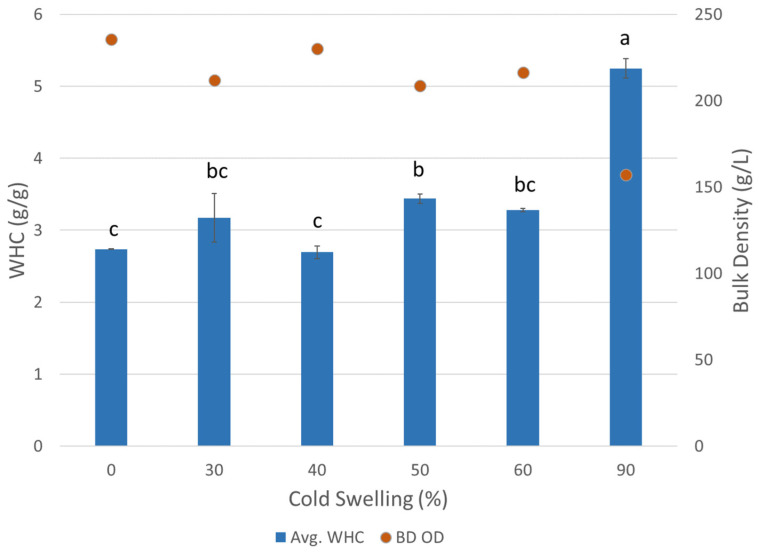
Water holding capacity (WHC) and off the dryer bulk density for extruded textured vegetable protein with different levels of cold-swelling proteins (0–90% CS). Error bars represent standard deviation. Different letters imply significant differences (*p* < 0.05).

The water holding capacity (WHC) is defined as the amount of water that is able to be absorbed and held by a material. The WHC is impacted by the internal structure of the extruded piece, as larger pores and internal open space allow for more water to be absorbed. Other protein functionality properties such as the protein type and ability to interact with water also affect the WHC, but the main contributor is the structure of the TVP formed during extrusion [16]. The fibrous structure of TVP is meant to mimic the texture of meat and be able to trap water inside its internal structure to create juiciness and tenderness like the myofibrillar structure of animal protein [14]. According to a previous study, soy- and wheat-based TVP showed a much higher WHC compared to freeze-dried meat from chicken, pork, and beef, with the WHC decreasing in that same order [14]. The same study also indicated that the WHC decreases as animal meat is cooked because the muscle structure is destroyed by thermal energy, and the fibrous structure is shrunk and densified so that it absorbs and holds less water. Therefore, a challenge arises with the TVP texture in that, to be similar to actual cooked meat, the WHC should be decreased. The WHC of TVP depends on several factors, such as the protein source, hydrophobicity, protein conformation structure, and extrusion processing parameters. Therefore, it was hypothesized that cold/heat-swelling characterization could be used to manipulate the structure of TVP and the WHC, as a lower % CS was predicted to lead to a lower WHC and a more densely layered product which would be most like cooked animal meat. Overall, there was an increasing trend in the WHC as the amount of cold-swelling proteins increased (Figure 9). However, significant differences between the intermediate treatments were minimal, and the only concrete differences in the WHC existed between 0, 50, and 90% CS. This means that the swelling properties of the proteins did impact the WHC, but only at the extreme ends, and the impact is minimized when both heat- and cold-swelling proteins are combined. The bulk density, as measured from the mass of extrudate filling a 1 L volume cup, was expected to trend inversely with respect to the WHC, as both are related to the internal structure of the extruded TVP. A less puffed and more compact TVP structure with higher bulk density would tend to hold less water, and this was found to be correct (Figure 9). There was an overall slight decreasing trend in bulk density as the % CS increased. Similar to the WHC results, the bulk density results indicate that the most significant differences exist between the most extreme treatments, 0 and 90% CS, with a slight trend shown for the intermediate treatments that would need to be confirmed by further research.

### 3.9. Texture Analysis

**Figure 10:**
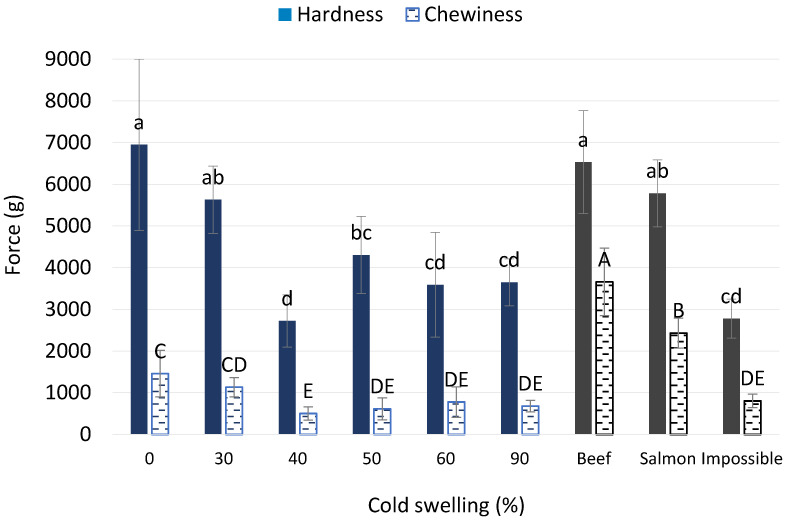
Texture profile analysis: hardness and chewiness of plant-based patties based on extruded TVP with different levels of cold-swelling proteins (0–90%). Error bars represent standard deviation. Different letters imply significant differences (*p* < 0.05).

The results from the TPA test on hydrated whole extrudate pieces produced no significant differences in peak force. This is thought to be caused by the variety of piece sizes and shapes that make it difficult to equally test each treatment and compare them amongst each other. Moreover, because the probe was smaller in diameter than the dish used to hold the sample, there were effects from the product escaping on the outer edges of the probe during compression. Compression testing of extruded pieces also is not the best representative of the texture of the actual product, as it is not in its final form. Therefore, plant-based burger patties made using the TVP from each treatment were tested instead to quantify the texture of the final product as would be experienced by consumers while eating. The TPA testing performed on plant-based patties was able to identify several significant differences and a clear decreasing trend in hardness (peak force) and chewiness as the amounts of cold-swelling proteins were increased (Figure 10). A higher % CS or CS:HS protein ratio led to an increased degree of crosslinking and greater porosity and lower bulk density, as discussed previously. This, in turn, led to a higher WHC. The hardness (and also chewiness) data align well with WHC and bulk-density trends, as a lower WHC and higher bulk density and greater structural compactness lead to an increase in the hardness of the TVP, which was consistent with results reported previously [16]. The only outlier to the linear decreasing trend in hardness was the 40% CS treatment. This could be caused by the functional soy proteins overpowering the wheat gluten used in the formulation at this ratio or because this treatment had a higher amount of soy flour (20%) than other mid-range treatments, such as the 50% and 60% CS, that only used 10% soy flour. Soy flour contains higher amounts of starch that gelatinizes as the sample is heated and causes an increase in viscosity. There was also a decreasing trend for chewiness as the % CS increased that was very comparable to the trend seen for hardness. This aligns with a previous study that showed that the integrity (resistance to being destroyed by high pressure, temperature, and shear) displayed an increasing trend as the solubility of the raw-material plant proteins decreased [14]. A previous study also reported a positive correlation between hardness and chewiness that corresponded with a lower WHC, leading to a firmer and chewier texture [16]. This means that a lower solubility protein source such as wheat, which is characterized as heat swelling, would cause a tougher and, therefore, chewier product than a high-solubility protein, which would most likely have cold-swelling functionality properties, as indicated by a high WAI.

### 3.10. Visual Analysis

**Figure 11:**
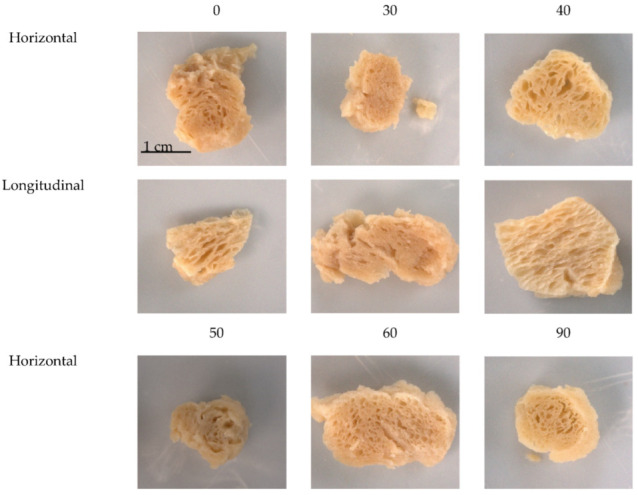

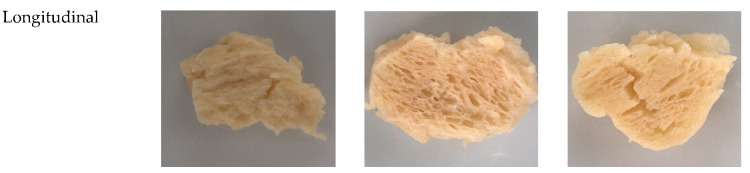
The internal structure of hydrated whole pieces of extruded TVP captured in the horizontal (perpendicular to flow exiting the extruder) and longitudinal directions (parallel to the flow) for products with different cold-swelling-protein concentrations (0–90% CS).

When looking closely at the horizontally cut pieces of TVP, the lower cold-swelling treatments, such as 0% CS, show a more layered appearance, with larger air cells, as was hypothesized (Figure 11). This is because treatments with more heat-swelling proteins, including wheat gluten, have the ability to form thin elastic protein films that are critical for the formation of a fibrous structure, along with a native fibrous structure instead of globular-like pea or soy [1]. Gluten contains gliadins and glutenins that form intramolecular and intermolecular disulfide bonds, respectively. The ratio of gliadins to glutenins in wheat gluten can, therefore, greatly impact functionality and texturization. Protein isolation, modification, extrusion, or other processing steps can modify the gluten subunits and structure and, therefore, greatly influence the functional properties of the protein that allow it to form a fibrous structure [24]. In comparison, TVPs at treatments larger than 50% CS shift to a more sponge-like structure with smaller pores, with less visible layering. Treatments higher in % CS contain larger amounts of soy protein isolate and pea protein isolate. Soy protein is known to have strong gelling abilities and to texturize very well. Pea protein also has gel-forming abilities, but these abilities are weaker than those of soy [24]. These gelling advantages influence the texture and structure of TVP by allowing the material to stretch and form a strong film so that the final TVP is expanded, with a porous internal matrix [1].

When looking at the longitudinal direction of the cut TVP pieces, the differences are more difficult to differentiate because all the pieces have visible layering, as this was the direction that the material was flowing out of the extruder. However, differences can be identified, as the lower % CS treatments show a more densely packed arrangement of layers. The shape of the pieces also changed as cold-swelling proteins increase, with the low-cold-swelling treatments (0% and 30%) lending a more irregular and jagged appearance to the pieces compared to other treatments that produced a more rounded and smooth outer appearance. As the % CS increases, there is also a slight increase in the paleness of the extrudates. This is because of the higher die temperatures required for heat-swelling proteins and the composition of amino acids, including increased lysine, that impacts the degree of Maillard reaction that is taking place [14]. Although extrusion can form TVP with a fibrous structure with many plant protein ingredients and sources, there are often still differences between the TVP’s structure and that of an actual cooked meat product. This is due to the differences in protein structure; gel formation; and amount, shape, and size of air cells. This can lead to visual and sensory differences in internal structure and affect texture parameters, such as the springiness, juiciness, and chewiness, along with the WHC and bulk density [14].

### 3.11. Sensory Analysis

Feedback from the focus groups provided valuable insights that can be used to guide changes in the design of the consumer sensory study. The recipes for the plant-based patties are described in Table 2. The focus group that tested the chewier/harder-textured low-CS patty (Group 1; beef hamburger control) had several positive reactions to the plant-based product, including the texture and how it did not crumble when chewing. Participants also liked the appearance of the sample because it was brown and crispy like real meat. Negative perceptions of the product were that the flavor was too bland, the product had an unidentifiable aroma, and there was a lack of mouthfeel from the absence of fat. Overall, participants liked the product enough that they would purchase it if it was a healthier and affordable option compared to actual meat. Many participants in the group found the product to be more like a pork sausage and would use the product in a breakfast-sandwich application at home.

The softer-textured high-CS focus group (Group 2; salmon patty control) had fewer positive results because the appearance and flavor were not thought to be fish-like. The texture was also described as being chewier and spongier than they would expect for a fish product. Some positive reactions included the aroma, the appearance of being plant-based, and moistness. In general, the participants were hesitant to accept a plant-based fish product because fish is thought to already be a healthier alternative to other meats. Overall, it was interesting to note that affordability, nutrition, and taste were the primary criteria in the evaluation of the plant-based meat products, even though environmental reasons are often touted as one of the drivers. Based on the feedback from the focus groups, the consumer study used only a beef hamburger as the control and focused on differences in liking and texture between several plant-based meat-patty treatments. The concentration of seasonings and oils was also increased to improve the flavor and mouthfeel of the plant-based burgers.

**Figure 12:**
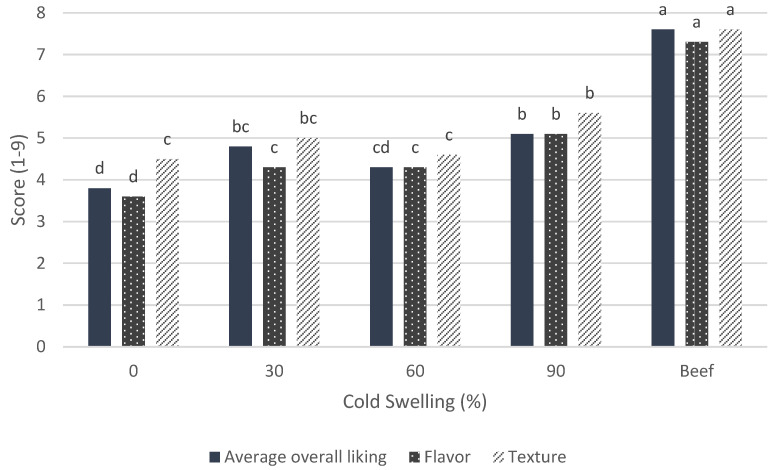
The average overall-liking scores from the consumer study for plant-based meat patties with different levels of cold-swelling proteins (% CS) and beef hamburger. Different letters imply significant differences (*p* < 0.05).

The consumer-study results for overall liking indicated that the most liked test sample was the softest treatment (90% CS; score of 5.1) and the least liked test sample was the firmest treatment (0% CS; score of 3.8) (Figure 12). Other factors that influence overall liking, such as flavor, texture, and appearance, were also tested and can be used to help identify the drivers of the overall-liking scores. The 0% CS treatment contained soy and wheat, the 90% CS contained soy and pea, and the other two intermediate treatments (30% and 60% CS) were only soy based. This could be the reason for the differences in overall liking because the flavor and aftertaste rankings also placed 90% CS as the most acceptable and 0% CS as the least acceptable. Therefore, there is evidence that the soy-and-pea flavor combination is more desirable than the soy-and-wheat combination. The 0% CS treatment also had high percentages of participants (37% and 50%) who thought that the flavor was either not strong enough or too strong, respectively. This unbalanced flavor profile is undesirable and could be due to the wheat/soy combination of the proteins used in this treatment, as discussed above, and might have contributed to the least overall liking for this treatment.

When looking at the overall scores for texture, we see that the 30% CS treatment is the highest (5.59) of the plant-based burgers, and 0% CS was the lowest (4.5). This could mean that a firm texture can be desirable but that 0% CS might be too firm. As shown by the TPA, the 0% CS treatment was the chewiest of the products, so the dislike of the texture of 0% CS could be because it was too chewy and not necessarily about the firmness. It could also mean that soy-based burgers have a desirable texture but that the soy flavor is too strong and influences the overall perception and liking by consumers and, therefore, reduces the overall-liking score. Further consumer studies are needed in the future that focus on individual ingredients to determine if the flavor of the specific plant source is in fact impacting the flavor-liking and overall-liking scores. Each plant-based treatment also had more participants rank the texture as too soft versus very few who thought it to be too firm.

When participants were asked about their overall perceptions and expectations for plant-based meat products, the most common benefits or reasons for purchasing included health benefits, affordability, and having a variety of flavor and meal options. The most mentioned concerns about plant-based meat were taste, texture, cost, and being highly processed, in that order.

## 4. Conclusions

This research aimed to understand how protein functionality attributes, such as swelling ability, can be used to manipulate and control plant-based meat textures. Plant proteins were characterized into two categories, heat swelling or cold swelling, and these were used to formulate meat analogue products targeting specific textures. It was hypothesized that including more cold-swelling proteins in a formulation would increase the softness of the product, while increasing heat swelling would cause an increase in firmness. This hypothesis was proven partially correct. A higher % CS or CS: HS protein ratio led to an increased degree of crosslinking and greater porosity and lower bulk density. This, in turn, led to a higher water holding capacity in the final product. A higher WHC and lower bulk density and less structural compactness or layering caused a decrease in the hardness of the product, ultimately leading to an increase in consumer liking. Overall, this research contributes to the understanding of protein functionality and proposes a novel technique to manipulate and control plant-based meat textures. This can have potential benefits for industry because it will allow for the creation of unique or previously unattainable textures, provide a method for quality control, and allow ingredients with similar functionality to be easily switched to address cost or supply-chain concerns. According to the consumer-study and focus-group results, texture and flavor are among several of the top consumer concerns when it comes to plant-based meat. Plant-based meat products have the potential to be a sustainable and nutritious protein alternative, but these hurdles need to be overcome to increase consumer acceptance.

## Figures and Tables

**Table 1:** Formulation details for each treatment. The % CS refers to different cold-swelling-to-heat-swelling or CS:HS protein ratios (example, 30% CS implies 30:70 CS:HS).

Cold Swelling (%)	0% CS	30% CS	40% CS	50% CS	60% CS	90% CS
Soy protein isolate		20	20	10	30	30
Soy protein conc. (Arcon F)	40	50		30	30	
Soy protein conc. (Arcon S)		10	20		30	30
Pea protein isolate				40		30
Vital wheat gluten	40		40			
Soy flour	20	20	20	10	10	10
Tapioca starch				10		
Protein content (%)	67	69.7	72.4	66.7	74.9	78.2

**Table 2:** Recipe for plant-based patties based on extruded texturized vegetable protein (TVP).

Ingredient	Description	(%)
TVP	Texturized vegetable protein (TVP) granules or shred made from soy, wheat, or pea	23.4–26.3
Water	Water or broth	55.2–58.5
Binder	Methylcellulose, Faba bean protein	8.9–9.5
Flavor	Low-CS protein patty: spices, yeast flakes, porcini powder, soy sauce, Worcestershire sauce, and liquid smoke High-CS protein patty: spices, dried roasted seaweed, soy sauce, miso paste, and yeast flakes	3–6
Color	Beet powder	0–0.7
Lipids	Coconut oil and vegetable oil	2–2.3

**Table 3:** Pilot-scale extrusion-parameter treatments with varying amounts of cold-swelling proteins (0–90% CS); SME = specific mechanical energy input, and IBM = in-barrel moisture content. Different letters in the same column indicate significant differences (*p* < 0.05).

CS (%)	SME (kJ/kg)	IBM (% wb)	Die Temp. (°C)
0	907.2 ^a^	42.5	161
30	997.2 ^a^	47.9	166
40	885.6 ^ab^	43.5	153
50	763.2 ^b^	37.9	159
60	936.0 ^a^	46.6	162
90	961.2 ^a^	39.6	153

**Table 4:** Least gelation concentration (LGC) for individual ingredients and raw-material blends with varying amounts of cold-swelling proteins (0–90% CS). Different letters imply significant difference (*p* < 0.05).

Treatment	Avg LGC
0% CS	17 ^ab^
30% CS	18 ^a^
40% CS	17 ^ab^
50% CS	15 ^ab^
60% CS	15 ^ab^
90% CS	14 ^ab^
SPI	11 ^b^
Arcon S	11 ^b^
Arcon F	13 ^ab^
GLU	13 ^ab^
PPI	15 ^ab^
Soy flour	17 ^ab^

**Table 5:** Protein solubility for raw-material blends and corresponding extruded treatments with varying amounts of cold-swelling proteins (0–90% CS). Different letters imply significant differences (*p* < 0.05).

% CS	Sample	Protein Solubility (%)
0	Raw	20.036 ^d^
0	Extruded	6.680
30	Raw	29.406 ^c^
30	Extruded	11.849
40	Raw	38.766 ^b^
40	Extruded	13.949
50	Raw	25.972 ^c^
50	Extruded	17.870
60	Raw	35.260 ^b^
60	Extruded	7.619
90	Raw	46.196 ^a^
90	Extruded	7.954

## Data Availability

The datasets generated for this study are available on request to the corresponding author.
